# Transportation: De-icers Add Sweet to Salt

**Published:** 2008-11

**Authors:** David Tenenbaum

With the onset of winter, towns are laying in a supply of road salt in preparation for icy weather. According to the U.S. Geological Survey’s *Mineral Commodity Summaries 2008*, the United States used nearly 50 million metric tons of salt in 2007, with 37% of that going toward road de-icing. Road salt—primarily sodium chloride, the cheapest option—is a lifesaver on slick roads, but it is also a growing cause of environmental contamination. So some companies are exploring ways to reduce how much de-icer it takes to keep roads clear.

A study by Eric V. Novotny and colleagues, slated for the 15 November 2008 issue of *Science of the Total Environment*, showed that seasonal and long-term changes in salinity in Minneapolis-area lakes closely followed the amount of road salt purchased by the state of Minnesota. In two of the lakes studied, a saline layer formed at the lake bottom and prevented oxygen transfer to the lake bed for months at a time, posing a potential threat to aquatic life. An earlier study, published by Domenico Sanzo and Stephen J. Hecna in volume 140, issue 2 (2006) of *Environmental Pollution*, found low survival, reduced weight, and abnormalities in frog larvae exposed to water that the authors said contained “environmentally realistic concentrations” of sodium chloride.

The U.S. Environmental Protection Agency (EPA) does not regulate road salt but acknowledges that special consideration and best management practices are needed to protect reservoirs and other drinking water supplies near treated highways and salt storage sites from contamination with road salt runoff, says spokesperson Dale Kemery.

Besides its environmental effects, road salt also has functional drawbacks: it easily washes away and must be reapplied, and sodium chloride can melt snow and ice in temperatures only down to about 16°F. But when sugar is mixed with salt, the combination can address both drawbacks, making the salt stick better and allowing it to work at lower temperatures, thus reducing the total need for salt.

One such product, Ice B’Gone, is made by Sears Ecological Applications Company of Rome, New York, and combines magnesium chloride (a salt) with “distillers condensed solubles” (a sugar byproduct obtained from molasses factories). Sears sells its additive to salt companies, which blend it with rock salt. Company president David Wood says the treated salt can be spread by highway agencies using existing equipment.

Ice B’Gone drops the minimum melting temperature to −45°F, Wood says, and although the treatment does raise the price of a ton of salt, it enables each ton to go further, netting a 25–40% reduction in road salt usage. In addition, he says, the Ice B’Gone formula reduces the corrosivity of the salt by as much as 70% over regular road salt. In 2008, that potential for reducing salt usage and associated corrosion earned Sears Ecological Applications the right to carry the EPA’s Design for the Environment label.

Sugar derived from sugar beets is the basis for another road salt additive called GeoMelt, made by SNI Solutions of Geneseo, Illinois. Mixed with rock salt, GeoMelt lowers the melting temperature to −30°F. The Ohio Department of Transportation tested GeoMelt in 11 counties last winter. The department’s deputy director, Scott Varner, says GeoMelt helped salt adhere better to the pavement and reduced the melting temperatures, and the state plans to continue evaluating the costs and benefits.

The 50% increase in price Ohio is paying for salt over the past year may tilt the tables in favor of additives, Varner says, because that raises the value of each ton saved by the additive. “If there are ways for us to use less salt, and it’s cheaper for our budgets,” he says, “ultimately that is good for the environment as well.”

